# Ozoralizumab shows effectiveness regardless of baseline RF and ACPA titres in patients with RA: a *post hoc* analysis of the OHZORA trial

**DOI:** 10.1093/rheumatology/keaf171

**Published:** 2025-03-26

**Authors:** Ryu Watanabe, Yoshiya Tanaka, Tsutomu Takeuchi, Shunsuke Okamoto, Masanao Kyuuma, Rumiko Matsumoto, Yoichi Nakayama, Masao Katsushima, Motomu Hashimoto

**Affiliations:** Department of Clinical Immunology, Osaka Metropolitan University Graduate School of Medicine, Osaka, Japan; The First Department of Internal Medicine, School of Medicine, University of Occupational and Environmental Health Japan, Kitakyushu, Japan; Keio University School of Medicine, Tokyo, Japan; Saitama Medical University, Saitama, Japan; Taisho Pharmaceutical Co, Ltd, Tokyo, Japan; Taisho Pharmaceutical Co, Ltd, Tokyo, Japan; Taisho Pharmaceutical Co, Ltd, Tokyo, Japan; Department of Rheumatology and Clinical Immunology, Kyoto University Graduate School of Medicine, Kyoto, Japan; Department of Clinical Immunology, Osaka Metropolitan University Graduate School of Medicine, Osaka, Japan; Department of Clinical Immunology, Osaka Metropolitan University Graduate School of Medicine, Osaka, Japan

**Keywords:** NANOBODY^®^, ozoralizumab, RA, RF, tumour necrosis factor-α, inhibitor, VHH antibody

## Abstract

**Objectives:**

Ozoralizumab (OZR) is a next-generation anti-TNF NANOBODY^®^ compound. The primary objective was to evaluate the efficacy of OZR in patients with RA in varying RF and anti-citrullinated peptide antibody (ACPA) titres. The secondary objective was to evaluate the changes in RF and ACPA titres following OZR treatment.

**Methods:**

A *post hoc* analysis was conducted on data from the Phase II/III OHZORA trial, which included 381 Japanese patients with RA who were treated with either 30 or 80 mg OZR over 52 weeks after demonstrating an inadequate response to MTX. Patients were classified into four groups based on the baseline RF or ACPA titre quartiles. The disease activity scores, RF and ACPA titres, plasma OZR concentrations and OZR-neutralizing antibody levels were evaluated. Statistical analyses included the Kruskal–Wallis test, two-way ANOVA and correlation analysis.

**Results:**

Treatment with OZR 30 mg significantly reduced disease activity in all the groups (*P* < 0.001), and the reduction in disease activity scores was comparable among the groups. Treatment with OZR 30 mg decreased the mean RF and ACPA titres from 149.5 to 71.1 IU/ml (*P* < 0.001) and 299.6 to 237.6 U/ml (*P* < 0.001), respectively. Effective trough concentrations of OZR were maintained for up to 52 weeks in all the groups, and baseline RF and ACPA titres were not associated with the generation of OZR-neutralizing antibodies.

**Conclusion:**

OZR is an effective anti-TNF treatment for RA, regardless of RF and ACPA titres, and exhibits promise as a novel therapeutic option.

## Graphical abstract

**Figure:**
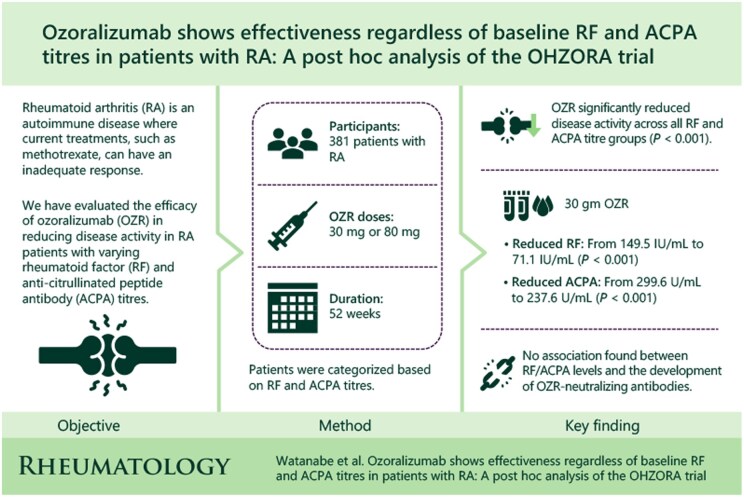

Rheumatology key messages:

- Ozoralizumab, a novel NANOBODY^®^ anti-TNF inhibitor, is effective regardless of RF and ACPA titres.
- Treatment with ozoralizumab decreases RF and ACPA titres over 52 weeks in RA patients.
- Ozoralizumab maintains effective plasma concentrations across varying RF and ACPA titre groups.

## Introduction

Tumour necrosis factor (TNF) is a cytokine with pleiotropic effects on various cell types and plays a central role in the pathogenesis of RA [1]. Among biologic disease-modifying antirheumatic drugs (bDMARDs), anti-TNF inhibitors are the most widely used bDMARDs, with long-term efficacy and safety well established [2]. Ozoralizumab (OZR) is a next-generation anti-TNF inhibitor which is a NANOBODY^®^ compound [3, 4]. The NANOBODY^®^ compound is a novel and unique class of antigen-binding fragments, derived from variable heavy-chain domains of heavy-chain antibody (VHH) [5]. OZR has a trivalent structure consisting of two anti-human TNF and one anti-human albumin NANOBODY^®^ compound. The Phase II/III OHZORA trial evaluated the efficacy and safety of OZR in patients with active RA despite MTX therapy [6, 7]. The study demonstrated that OZR was well-tolerated and effectively controlled disease activities, thus OZR (30 mg every 4 weeks) became the first NANOBODY^®^ compound approved for the treatment of RA in Japan [4].

Generally, the efficacy of anti-TNF antibodies containing the fragment crystallizable (Fc) portion is reduced in patients with high titres of RF [8, 9]. For instance, RF-negative patients demonstrated greater improvements in disease activity scores compared with RF-positive patients when treated with these agents [9]. Moreover, non-responders to these therapies exhibited significantly higher RF levels than responders [8]. In contrast, certolizumab pegol (CZP), which lacks the Fc portion, has demonstrated efficacy irrespective of RF titres in both clinical trials and real-world clinical settings [10, 11]. This may be attributed to the immune complex formed by RF pentamer and anti-TNF antibody with the Fc portion that is phagocytosed via Fc receptors expressed on macrophages in patients with high RF titres, which may reduce the blood concentration of the anti-TNF antibody [12, 13]. However, the efficacy of OZR, which lacks the Fc portion in patients with high RF titres, remains unclear.

In this *post hoc* analysis of the OHZORA trial, we aimed to assess whether the efficacy of OZR was influenced by the titres of RF. In addition, we aimed to evaluate changes in RF and anti-citrullinated peptide antibody (ACPA) titres in patients with RA treated with OZR.

## Methods

### Study design

In this study, we aimed to evaluate the efficacy of OZR in patients with varying RF and ACPA titres as the primary objective and to assess changes in RF and ACPA titres following OZR treatment as the secondary objective. To achieve this, a *post hoc* analysis of the OHZORA trial was conducted, and data from the trial were retrospectively analysed. The study design of the trial has been previously described [7]. Briefly, the OHZORA trial (JapicCTI-184029) was a multicentre, randomized, placebo-controlled, double-blind, parallel-group confirmatory trial consisting of a 24-week double-blind treatment period (period A) followed by a 28-week open-label period (period B) to evaluate the efficacy and safety of long-term OZR treatment. A total of 381 patients were allocated to the placebo (*n* = 75), OZR 30 mg (*n* = 152) or OZR 80 mg (*n* = 154) groups at baseline. The placebo group was reallocated to the OZR 30 mg (*n* = 44) or 80 mg (*n* = 23) groups depending on whether the early escape criteria were met between weeks 20 and 24. The OZR 30 mg group was reallocated to the 80 mg group only if they met the early escape criteria (*n* = 9) at week 20. Thus, 143 patients continuously received OZR 30 mg over 52 weeks. The OZR 80 mg group allocated at baseline continued treatment for 52 weeks (*n* = 154).

### Patients

The OHZORA trial included Japanese patients with RA aged 20–75 years who exhibited an inadequate response to MTX. The inclusion criteria and exclusion criteria have been previously described [7].

### Assessments

The efficacy endpoints have been previously described [7]. Disease activity score in 28 joints using CRP or ESR (DAS28-CRP/ESR), Clinical Disease Activity Index (CDAI), Simplified Disease Activity Index (SDAI) and titres of RF and ACPA were evaluated at each visit. Plasma OZR concentrations and plasma OZR-neutralizing antibodies were measured as previously described [7].

### Ethics approval

The OHZORA study was conducted in accordance with the Declaration of Helsinki, and all study sites received approval from ethical committees (e.g. Doujin Memorial Medical Foundation, Meiwa Hospital institutional review board). Written informed consent to participate in the study was obtained from all patients.

### Statistical analysis

The clinical characteristics of patients were compared using the Kruskal–Wallis nonparametric test for continuous variables and Pearson’s *χ*^2^ test for categorical variables. The last observation carried forward (LOCF) method was used for disease activity assessment of missing data, including discontinuation. DAS28-CRP, DAS28-ESR, CDAI, SDAI and titres of RF and ACPA were compared by Wilcoxon matched-pairs signed rank test. The four groups were compared using two-way ANOVA with Tukey’s multiple comparison test. Drug retention was analysed using the Kaplan–Meier method and compared using the log-rank test. Correlations were examined using Spearman’s correlation analysis. Statistical analyses were performed using EZR (Saitama Medical Center, Jichi Medical University, Saitama, Japan), a graphical user interface for R software (R Foundation for Statistical Computing, Vienna, Austria) [14] or GraphPad Prism 10 (GraphPad Software Inc., La Jolla, CA, USA), as appropriate. A *P*-value <0.05 in a two-sided test was considered statistically significant.

## Results

### Clinical characteristics of patients according to baseline RF titres

A total of 381 patients were classified into four groups according to the baseline RF titre quartiles (RF1: RF 3–20 IU/ml, RF2: 20–49 IU/ml, RF3: 49–153 IU/ml, RF4: 153–2029 IU/ml). The baseline characteristics of the four groups are shown in Supplementary Table S1, available at *Rheumatology* online. Patients with higher RF levels were older and had a longer disease duration, higher ACPA titres, higher ESR levels and higher disease activity. The analysis was limited to 143 patients who continuously received OZR 30 mg over 52 weeks (RF1, *n* = 31; RF2, *n* = 40; RF3, *n* = 37 and RF4, *n* = 35), which is the approved dose in Japan. Patients with higher RF levels had a longer disease duration and higher ACPA titres, higher ESR and hs-CRP levels, higher disease activities and higher interleukin (IL)-6 and matrix metalloproteinase-3 levels at baseline. However, the concomitant MTX dose, glucocorticoid (GC) usage, and prior biologics use were comparable among the four groups (Table 1).

**Table 1.** Baseline characteristics of 143 patients who received ozoralizumab 30 mg classified into four groups based on RF titres

Variable	RF1 (3 ≤ RF < 20)	RF2 (20 ≤ RF < 49)	RF3 (49 ≤ RF < 152)	RF4 (152 ≤ RF)	*P*-value
*N*	31	40	37	35	
Age, years	56.00 [48.00, 64.50]	53.00 [45.75, 61.00]	56.00 [48.00, 66.00]	56.00 [49.00, 63.00]	0.486
Female sex, %)	19 (61.3)	33 (82.5)	27 (73.0)	20 (57.1)	0.076
Disease duration, years	2.00 [1.00, 6.00]	6.00 [1.00, 8.00]	8.00 [3.00, 12.00]	7.00 [4.00, 10.00]	0.01
RF-positive, %	8 (25.8)	40 (100.0)	37 (100.0)	35 (100.0)	<0.001
RF, IU/ml	8.00 [3.00, 15.50]	26.50 [21.75, 32.25]	71.00 [62.00, 109.00]	377.00 [247.00, 490.50]	<0.001
ACPA-positive, %	19 (61.3)	39 (97.5)	36 (97.3)	35 (100.0)	<0.001
ACPA, U/ml	13.30 [0.55, 120.50]	98.00 [34.65, 407.25]	135.00 [44.90, 392.00]	165.00 [84.15, 761.00]	<0.001
Seropositive, %	20 (64.5)	40 (100)	37 (100)	35 (100)	<0.001
RF & ACPA-positive, %	13 (41.9)	35 (87.5)	33 (89.2)	33 (94.3)	<0.001
ESR, mm/h	23.00 [16.00, 33.50]	30.00 [18.00, 44.00]	42.00 [27.00, 51.00]	53.00 [30.00, 74.00]	<0.001
hs-CRP, mg/dl	0.48 [0.27, 1.25]	0.52 [0.08, 1.67]	0.81 [0.44, 2.13]	1.62 [0.46, 3.64]	0.018
IL-6, pg/ml	12.10 [3.83, 37.45]	13.45 [2.94, 39.25]	17.80 [7.07, 52.60]	63.70 [8.01, 107.00]	0.003
MMP-3, μg/l	118.10 [81.80, 231.90]	123.50 [57.28, 232.92]	136.30 [52.30, 218.40]	205.90 [139.80, 265.85]	0.037
SJC28	9.00 [6.50, 11.50]	8.00 [7.00, 10.00]	9.00 [7.00, 11.00]	12.00 [7.00, 16.00]	0.093
TJC28	9.00 [6.50, 13.50]	9.00 [7.00, 13.00]	10.00 [8.00, 13.00]	12.00 [9.00, 16.00]	0.116
Pain VAS, mm	37.00 [17.50, 65.00]	43.00 [24.75, 67.25]	49.00 [36.00, 77.00]	69.00 [33.00, 84.00]	0.073
Ph-GA, mm	61.00 [42.50, 76.50]	62.00 [39.75, 71.25]	55.00 [45.00, 71.00]	70.00 [51.50, 78.50]	0.076
Pt-GA, mm	48.00 [23.00, 74.00]	49.50 [21.00, 76.25]	59.00 [36.00, 77.00]	63.00 [33.00, 84.00]	0.127
DAS28-CRP	4.62 [4.18, 5.65]	4.95 [4.17, 5.50]	5.32 [4.69, 5.74]	5.68 [4.60, 6.60]	0.013
DAS28-ESR	5.48 [4.87, 5.81]	5.56 [4.80, 6.26]	6.04 [5.38, 6.39]	6.54 [5.64, 7.30]	<0.001
CDAI	29.00 [23.00, 36.60]	26.50 [22.05, 34.35]	31.20 [27.00, 34.80]	37.00 [29.28, 47.25]	0.008
SDAI	29.30 [23.73, 38.00]	27.84 [23.55, 38.61]	32.68 [28.55, 35.88]	40.58 [30.59, 53.10]	0.004
HAQ-DI	0.88 [0.44, 1.12]	1.00 [0.47, 1.25]	1.00 [0.38, 1.50]	1.12 [0.81, 1.75]	0.244
MTX, mg/week	10.00 [7.00, 12.00]	11.50 [8.00, 12.00]	10.00 [8.00, 12.00]	10.00 [8.00, 12.00]	0.553
GC use, %	17 (54.8)	16 (40.0)	13 (35.1)	15 (42.9)	0.415
Prior biologics use, %	6 (19.4)	10 (25.0)	16 (43.2)	15 (42.9)	0.07

Results are expressed as median [interquartile range] for continuous variables or the number (%) for nominal variables. Prior biologics use includes prior use of any biologic and/or targeted synthetic disease-modifying antirheumatic drugs.

ACPA, anti-citrullinated peptide antibody; CDAI, clinical disease activity index; DAS28-CRP, disease activity score using CRP; DAS28-ESR, disease activity score using ESR; GC, glucocorticoid; HAQ-DI, health assessment questionnaire disability index; hs-CRP, high-sensitivity CRP; IL-6, interleukin-6; MMP-3, matrix metalloproteinase-3; Ph-GA, physicians’ global assessment; Pt-GA, patients’ global assessment; SDAI, simplified disease activity index; SJC28, swollen joint count of 28 joints; TJC28, tender joint count of 28 joints; VAS, visual analogue scale.

### Efficacy of OZR in the RF titre groups

**Figure 1.**
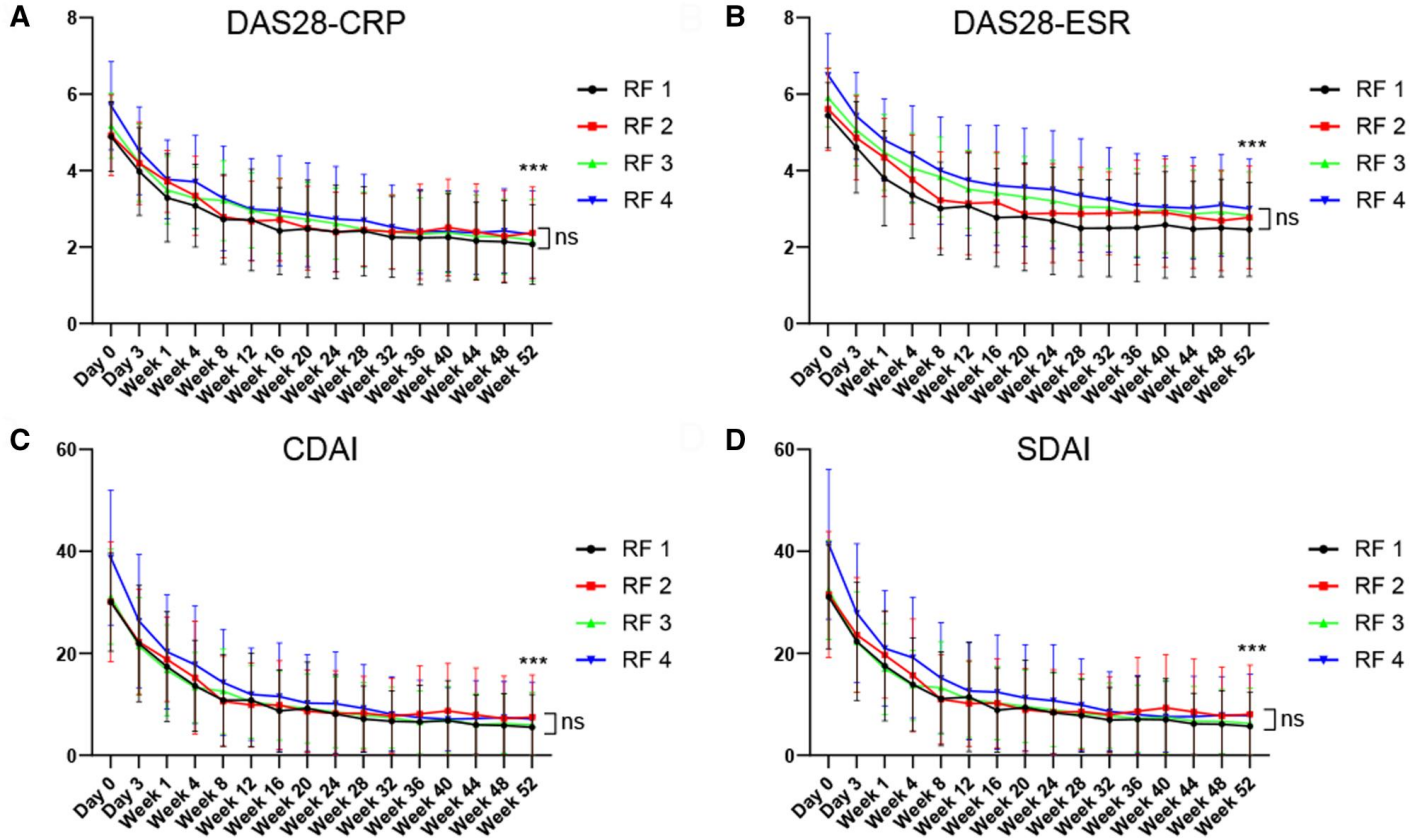
Ozoralizumab (OZR) 30 mg effectively controlled disease activities over 52 weeks irrespective of baseline RF titres. A total of 143 patients who received OZR 30 mg over 52 weeks were classified into four groups based on the baseline RF titre quartiles (RF1: RF 3–20 IU/ml, RF2: 20–49 IU/ml, RF3: 49–153 IU/ml, RF4: 153–2029 IU/ml), and changes in (A) DAS28-CRP, (B) DAS28-ESR, (C) CDAI and (D) SDAI were shown. Data are shown as mean ± SD. The last observation carried forward method was used. CDAI, clinical disease activity index; DAS28-CRP, disease activity score using CRP; DAS28-ESR, disease activity score using ESR; ns, not significant; SDAI, simplified disease activity index. ***P < 0.001

Despite the differences in baseline characteristics, OZR 30 mg effectively controlled disease activity in each RF titre group (Fig. 1). DAS28-CRP levels significantly decreased in all groups between baseline and 52 weeks (*P* < 0.001 for all groups, Fig. 1A). As a result, DAS28-CRP at 52 weeks was comparable among the four groups (*P* = 0.81). DAS28-ESR, CDAI and SDAI also significantly decreased in all the RF groups between baseline and at 52 weeks (*P* < 0.001 for all the groups). At 52 weeks, DAS28-ESR, CDAI and SDAI were not different among the four groups (*P* = 0.51, *P* = 0.69 and *P* = 0.64, respectively).

OZR 80 mg also effectively controlled disease activity over 52 weeks in each RF titre group (Supplementary Fig. S1, available at *Rheumatology* online). DAS28-CRP, DAS28-ESR, CDAI and SDAI significantly decreased in all the RF groups between baseline and 52 weeks (*P* < 0.001 for all the groups). Thus, DAS28-CRP, DAS28-ESR, CDAI and SDAI did not differ among the four groups at 52 weeks (*P* = 0.47, *P* = 0.50, *P* = 0.64 and *P* = 0.58, respectively). In contrast, disease activity slowly decreased in patients assigned to the placebo group (Supplementary Fig. S2, available at *Rheumatology* online). A comparison of disease activity at week 24 between OZR 30 mg and placebo demonstrated that the OZR 30 mg achieved significantly better disease control than placebo across all the RF titre groups (Supplementary Fig. S3, available at *Rheumatology* online). These results indicate that OZR 30 mg could be effective regardless of the baseline RF titre.

### Clinical characteristics of patients according to baseline ACPA titres

Then, we classified 381 patients into four groups according to baseline ACPA titre quartiles (ACPA1: ACPA 0.5–25.9 U/ml, ACPA2: 25.9–103 U/ml, ACPA3: 103–426 U/ml, ACPA4: 426–1200 U/ml). The baseline characteristics of the four groups are shown in Supplementary Table S2, available at *Rheumatology* online. Patients with higher ACPA levels were older, had higher RF titres, higher ESR levels and higher DAS28-ESR. The analysis was limited to 143 patients who received OZR 30 mg over 52 weeks (ACPA1, *n* = 32; ACPA2, *n* = 35; ACPA3, *n* = 45 and ACPA4, *n* = 31). Patients with higher ACPA levels were older and had higher RF titres and DAS28-ESR at baseline; however, the concomitant MTX dose, GC usage and prior biologics use were comparable among the four groups (Table 2).

**Table 2.** Baseline characteristics of 143 patients who received ozoralizumab 30 mg classified into four groups based on anti-citrullinated peptide antibody titres

Variable	ACPA1 (0.5 ≤ ACPA < 25.9)	ACPA2 (25.9 ≤ ACPA < 103)	ACPA3 (103 ≤ ACPA < 426)	ACPA4 (426 ≤ ACPA)	*P*-value
*N*	32	35	45	31	
Age, years	55.00 [44.00, 64.00]	55.00 [48.00, 61.75]	55.00 [46.00, 61.25]	59.00 [51.75, 66.00]	0.045
Female sex, %	67 (70.5)	81 (86.2)	71 (74.0)	66 (68.8)	0.026
Disease duration, years	5.00 [1.00, 9.00]	7.00 [2.00, 11.00]	5.00 [3.00, 11.00]	6.00 [1.00, 11.25]	0.285
RF-positive, %	18 (56.2)	33 (94.3)	39 (86.7)	30 (96.8)	<0.001
RF, IU/ml	15.00 [3.00, 37.50]	61.00 [22.25, 149.25]	61.00 [25.00, 178.75]	71.50 [35.50, 281.75]	<0.001
ACPA-positive, %	18 (56.2)	35 (100)	45 (100)	31 (100)	<0.001
ACPA, U/ml	1.90 [0.50, 10.85]	51.95 [35.00, 69.58]	193.00 [140.50, 323.25]	911.00 [570.25, 1200.00]	<0.001
Seropositive, %	21 (65.6)	35 (100)	45 (100)	31 (100)	<0.001
RF & ACPA-positive, %	5 (15.6)	34 (97.1)	44 (97.8)	31 (100)	<0.001
ESR, mm/h	32.00 [21.50, 38.50]	35.50 [26.00, 50.00]	37.00 [23.75, 50.25]	40.50 [26.75, 53.00]	0.009
hs-CRP, mg/dl	0.60 [0.18, 1.33]	0.86 [0.40, 1.83]	0.62 [0.20, 1.74]	0.81 [0.29, 2.31]	0.074
IL-6, pg/ml	13.00 [4.17, 44.20]	16.80 [6.68, 42.17]	17.20 [6.09, 67.42]	25.95 [7.25, 66.23]	0.112
MMP-3, μg/l	126.60 [57.35, 228.75]	145.45 [81.52, 225.73]	146.95 [80.95, 237.62]	151.75 [81.58, 247.07]	0.368
SJC28	8.00 [7.00, 13.00]	8.00 [7.00, 12.00]	9.00 [7.00, 11.00]	9.00 [7.00, 11.00]	0.945
TJC28	9.00 [6.00, 14.00]	9.50 [7.00, 13.00]	10.00 [7.00, 14.00]	10.00 [7.00, 15.00]	0.533
Pain VAS, mm	42.00 [22.50, 71.00]	46.00 [25.25, 74.00]	52.00 [30.75, 78.25]	61.00 [37.00, 77.25]	0.019
Ph-GA, mm	63.50 [44.75, 77.25]	59.50 [42.00, 71.75]	61.00 [43.75, 76.25]	65.00 [48.25, 76.00]	0.697
Pt-GA, mm	48.50 [25.25, 75.00]	48.50 [29.00, 69.75]	58.00 [30.75, 79.25]	60.00 [42.75, 80.00]	0.045
DAS28-CRP	5.00 [4.18, 5.73]	5.10 [4.44, 5.68]	4.99 [4.37, 5.75]	5.25 [4.68, 6.10]	0.053
DAS28-ESR	5.59 [4.92, 6.40]	5.68 [5.21, 6.46]	5.72 [5.15, 6.51]	6.06 [5.35, 6.77]	0.043
CDAI	31.90 [22.60, 41.20]	28.70 [23.20, 37.40]	31.00 [24.40, 37.25]	32.80 [24.50, 38.65]	0.373
SDAI	32.64 [23.94, 41.75]	29.93 [24.31, 38.25]	32.68 [25.04, 38.61]	34.90 [26.07, 42.87]	0.163
HAQ-DI	0.88 [0.38, 1.38]	1.12 [0.53, 1.38]	0.88 [0.50, 1.50]	1.13 [0.62, 1.50]	0.145
MTX, mg/week	10.00 [8.00, 12.00]	10.00 [8.00, 12.00]	10.00 [8.00, 12.00]	10.00 [8.00, 12.00]	0.721
GC use, %	37 (38.9)	42 (44.7)	43 (44.8)	41 (42.7)	0.833
Prior biologics use, %	34 (35.8)	37 (39.4)	31 (32.3)	30 (31.2)	0.636

Results are expressed as median [interquartile range] for continuous variables or the number (%) for nominal variables. Prior biologics use includes prior use of any biologic and/or targeted synthetic disease-modifying antirheumatic drugs.

ACPA, anti-citrullinated peptide antibody; CDAI, clinical disease activity index; DAS28-CRP, disease activity score using CRP; DAS28-ESR, disease activity score using ESR; GC, glucocorticoid; HAQ-DI, health assessment questionnaire disability index; hs-CRP, high-sensitivity CRP; IL-6, interleukin-6; MMP-3, matrix metalloproteinase-3; Ph-GA, physicians’ global assessment; Pt-GA, patients’ global assessment; SDAI, simplified disease activity index; SJC28, swollen joint count of 28 joints; TJC28, tender joint count of 28 joints; VAS, visual analogue scale.

### Efficacy of OZR in the ACPA titre groups

**Figure 2.**
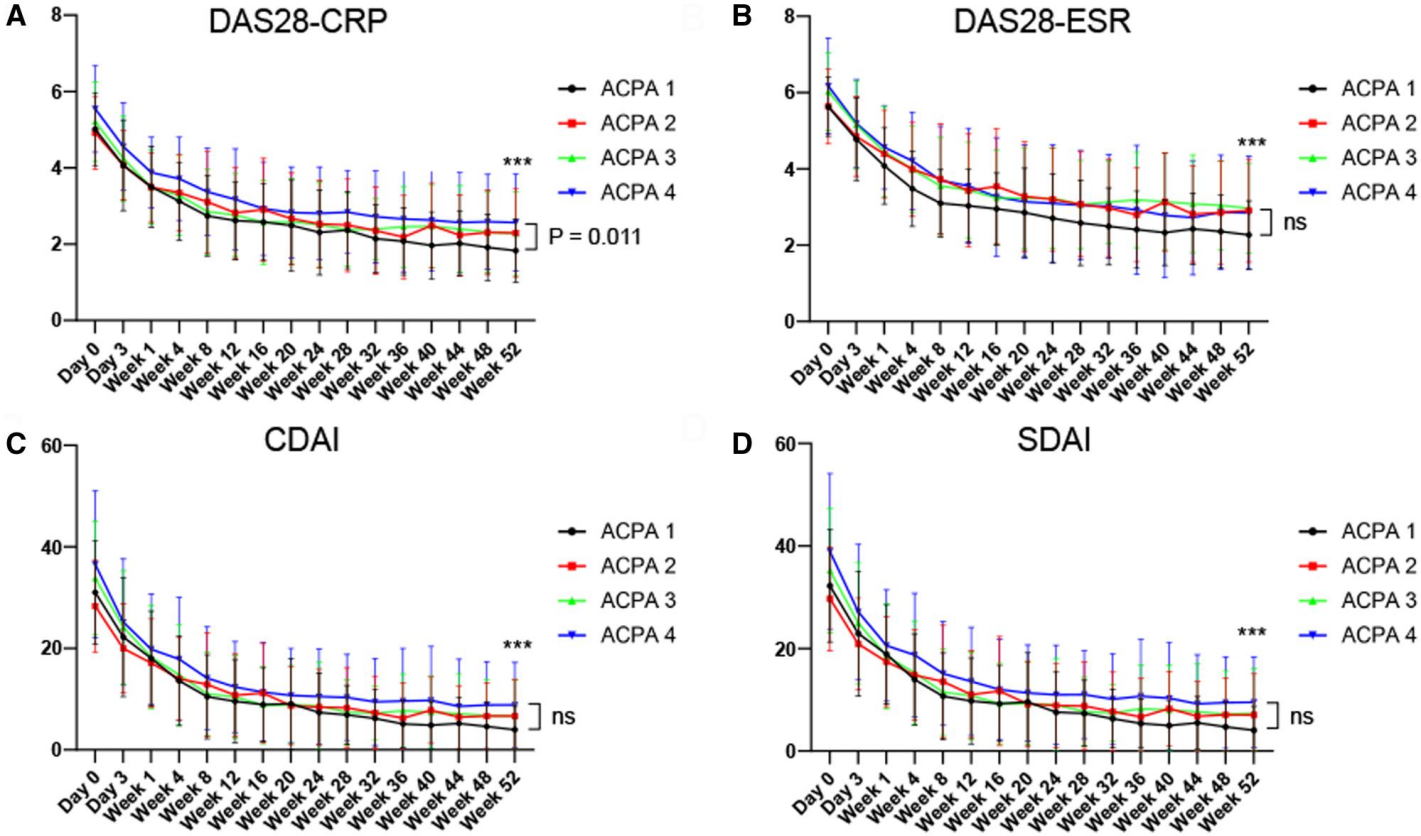
Ozoralizumab (OZR) 30 mg effectively controlled disease activities over 52 weeks irrespective of baseline anti-citrullinated peptide antibody (ACPA) titres. A total of 143 patients who received OZR 30 mg over 52 weeks were classified into four groups based on the baseline ACPA titre quartiles (ACPA1: 0.5–25.9 U/ml, ACPA2: 25.9–103 U/ml, ACPA3: 103–426 U/ml, ACPA4: 426–1200 U/ml), and changes in (A) DAS28-CRP, (B) DAS28-ESR, (C) CDAI and (D) SDAI were shown. Data are shown as mean ± SD. The last observation carried forward method was used. CDAI, clinical disease activity index; DAS28-CRP, disease activity score using CRP; DAS28-ESR, disease activity score using ESR; ns, not significant; SDAI, simplified disease activity index. ***P < 0.001

Although some differences in baseline characteristics existed, OZR 30 mg efficiently controlled disease activity in each ACPA titre group (Fig. 2). DAS28-CRP levels significantly decreased in all groups between baseline and 52 weeks (*P* < 0.001 for all the groups). At 52 weeks, DAS28-CRP levels were higher in the ACPA4 group than in the ACPA1 group (*P* = 0.011); however, changes in DAS28-CRP levels between baseline and 52 weeks were comparable among the four groups (*P* = 0.19). In addition, DAS28-ESR, CDAI and SDAI significantly decreased in all the groups between baseline and 52 weeks (*P* < 0.001 for all the groups), and changes in DAS28-ESR, CDAI and SDAI between baseline and 52 weeks were comparable among the four groups (*P* = 0.35, *P* = 0.41 and *P* = 0.39, respectively). Moreover, OZR 80 mg also effectively controlled disease activity over 52 weeks in each ACPA titre group (Supplementary Fig. S4, available at *Rheumatology* online). These results indicate that OZR could be effective, irrespective of the ACPA titre.

### Drug retention of OZR in the RF titre groups

The drug retention rates of OZR 30 mg were examined for each RF titre group (Supplementary Fig. S5A, available at *Rheumatology* online). The highest RF group (RF4) showed the lowest drug retention rate (*P* = 0.033). However, the nine discontinuations in the RF4 group were due to side effects in six patients (pleural bacterial infection, dyspnoea, lumber spinal stenosis, pneumonia, increase of blood β-D-glucan and retinal haemorrhage), other reasons in two patients, and withdrawal of consent in one patient. The ACPA titre did not significantly affect the drug retention of OZR 30 mg (Supplementary Fig. S5B, available at *Rheumatology* online). In patients treated with OZR 80 mg (Supplementary Figs S5C and D, available at *Rheumatology* online), RF and ACPA titres did not influence drug retention. These results suggest that RF titre does not necessarily have a consistent effect on OZR drug retention.

### Decrease of RF titre by OZR treatment

**Figure 3.**
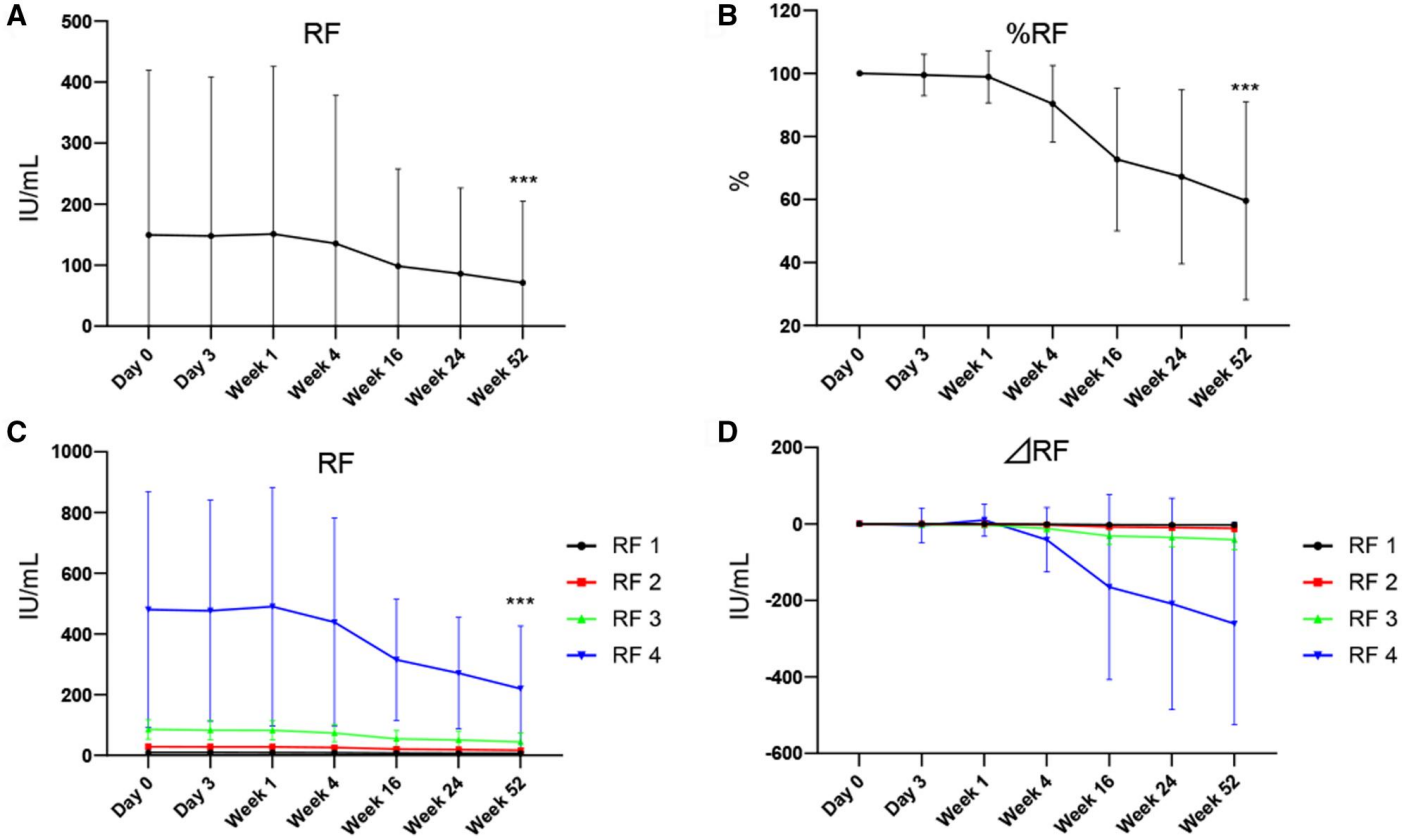
RF titre reduction by ozoralizumab (OZR) treatment. The mean change (A) and percent change (B) of the RF titre in patients who received OZR 30 mg. The mean change of RF titre (C) and RF reduction (D) in each RF titre group. Data are shown as mean ± SD. ***P < 0.001

The decrease of RF titre by OZR was examined (Fig. 3). Fig. 3A shows the mean change in RF titre. Treatment with OZR 30 mg decreased the mean RF titre from 149.5 to 71.1 IU/ml (*P* < 0.001). Fig. 3B shows the percent change in RF titre. OZR reduced the RF titre from 100% at baseline to 59.6% at 52 weeks (*P* < 0.001). Fig. 3C and D shows the mean change of RF titre and the reduction of RF titre from baseline in each RF titre group, respectively. The highest RF group (RF4) exhibited the largest RF titre reduction (*P* < 0.001).

### Decrease of ACPA titre by OZR treatment

**Figure 4.**
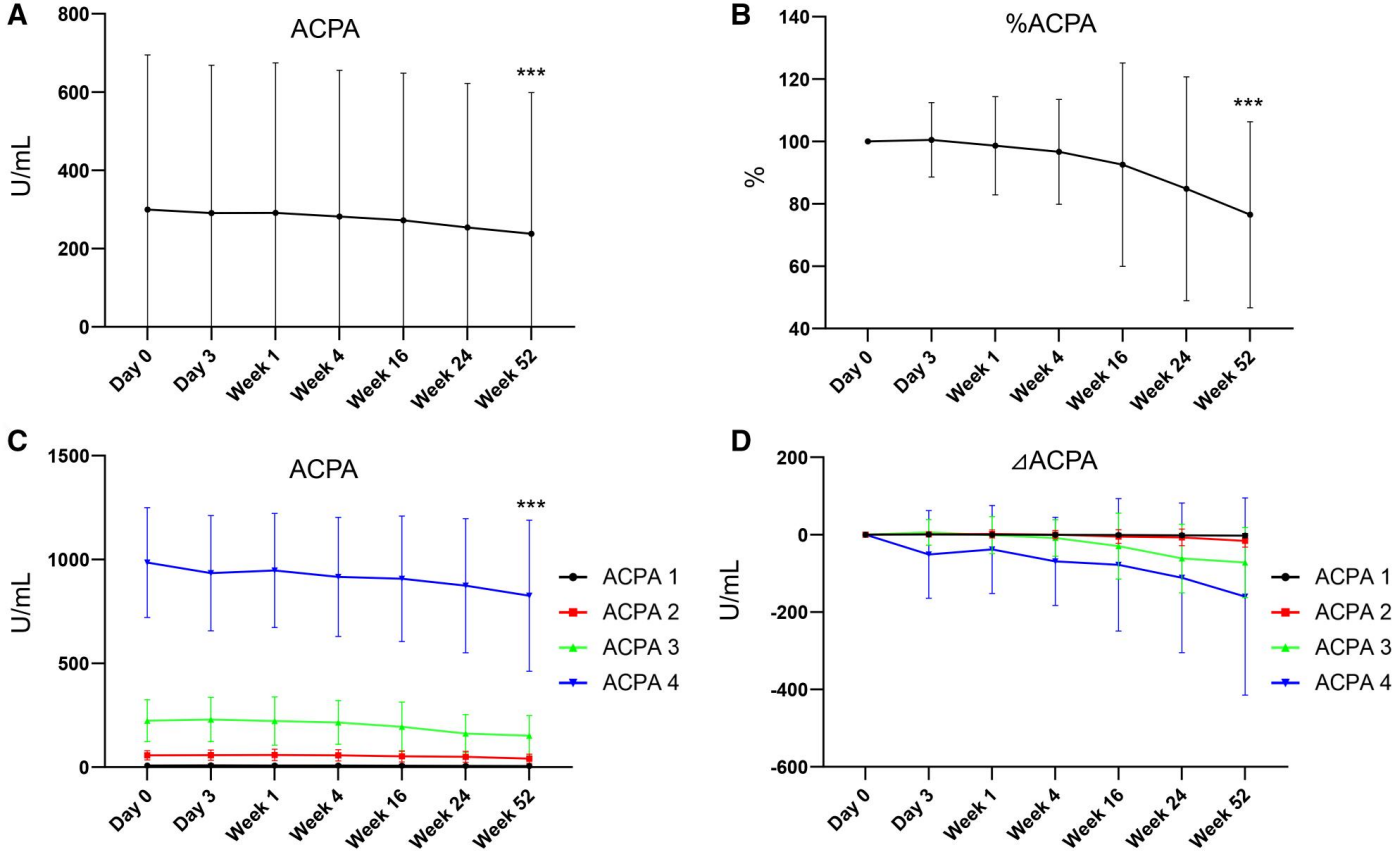
Anti-citrullinated peptide antibody (ACPA) titre reduction by ozoralizumab (OZR) treatment. The mean change (A) and percent change (B) of ACPA titre in patients who received OZR 30 mg. The mean change of ACPA titre (C) and ACPA reduction (D) in each ACPA titre group. Data are shown as mean ± SD. ***P < 0.001

The decrease of ACPA titre by OZR was examined (Fig. 4). Fig. 4A shows the mean change of ACPA titre. Treatment with OZR 30 mg decreased the mean ACPA titre from 299.6 to 237.6 U/ml (*P* < 0.001). Fig. 4B shows the percent change in ACPA titre. OZR reduced the ACPA titre from 100% at baseline to 76.5% at 52 weeks (*P* < 0.001). Fig. 4C and D shows the mean change of ACPA titre and the reduction of ACPA titre in each ACPA titre group, respectively. Similar to RF, the highest ACPA group (ACPA4) exhibited the largest ACPA titre reduction (*P* < 0.001).

### Correlation between RF and ACPA titres and serum IL-6 levels

Correlation between titres of RF and ACPA and serum IL-6 levels were examined (Supplementary Fig. S6, available at *Rheumatology* online), as IL-6 is known to promote autoantibody production [15]. RF titres, but not ACPA titres, showed a significant correlation with IL-6 levels at baseline. Furthermore, the reduction in RF titres, but not ACPA titres, from baseline to week 52 was significantly correlated with a decrease in IL-6 levels. These findings suggest that the reduction of IL-6 by OZR may contribute to the decrease in RF titres.

### Multivariable logistic regression analysis for DAS28-CRP remission

To identify factors associated with DAS28-CRP remission in 143 patients treated with OZR 30 mg, multivariable logistic regression analysis was performed (Supplementary Table S3, available at *Rheumatology* online). In Model 1, the RF and ACPA titre groups, MTX dose and prior biologic use were included as variables, while in Model 2, reductions in RF and ACPA titers, MTX dose and prior biologic use were included as variables. Not only baseline RF and ACPA titres but also reductions in RF and ACPA titres were not associated with remission. These findings indicate that RF and ACPA titres are not predictive factors for DAS28-CRP remission.

### Plasma OZR concentration in the RF and ACPA titre groups

The plasma OZR concentrations in each RF titre group were examined (Supplementary Fig. S7A, available at *Rheumatology* online). In each group, the plasma trough concentrations increased until week 16 and reached a plateau. The plasma OZR concentrations tended to be slightly lower in the highest RF titre group (RF4) than in the lowest group (RF1) at 52 weeks, but there was no significant difference between the two groups (*P* = 0.061). Similarly, the plasma OZR concentrations increased until week 16 in each ACPA titre group (Supplementary Fig. S7B, available at *Rheumatology* online). These concentrations were significantly lower in the higher ACPA titre group (ACPA3) than in the lowest group at 52 weeks (ACPA1, *P* = 0.031). These results indicate that plasma OZR levels tend to be slightly lower in patients with higher autoantibody levels.

### Plasma OZR-neutralizing antibodies

Finally, OZR-neutralizing antibodies in each RF titre group were examined (Supplementary Fig. S8, available at *Rheumatology* online). No consistent trend in the presence of OZR-neutralizing antibodies existed in all patients (*n* = 381) or in the OZR 30 mg group (*n* = 143). This was also observed in the ACPA titre group. These results indicated that baseline RF or ACPA titres did not affect the generation of plasma OZR-neutralizing antibodies.

## Discussion

In this *post hoc* analysis of the OHZORA study, OZR 30 mg demonstrated effectiveness in all the RF and ACPA titre groups. In addition, OZR 30 mg reduced RF and ACPA titres by 40% and 24%, respectively, after 52 weeks. Effective trough OZR concentrations were achieved and maintained for 52 weeks in all the RF and ACPA titre groups. These results suggest that OZR is a promising anti-TNF inhibitor and shows effectiveness regardless of the RF and ACPA titres. OZR could be an optimal drug in patients with inadequate response to MTX, particularly with high autoantibody titres.

In the early 1990s, heavy-chain antibodies were discovered in camelids [4]. Conventional antibodies recognize antigens with variable regions of the heavy and light chains, whereas heavy-chain antibodies recognize antigens with the variable region of the heavy chain alone [4]. VHH antibodies using variable heavy-chain domains of heavy-chain antibody have high tissue-penetrating capacity because of their low molecular weight [16]. Although the molecular size of conventional anti-TNF inhibitors is ∼150 kDa, that of OZR is 38 kDa, allowing rapid tissue distribution, particularly in inflamed joints [17]. In addition, anti-TNF inhibitors form immune complexes with TNFα. Compared with adalimumab-TNFα immune complexes, OZR-TNFα immune complexes are smaller and lack an Fc portion, which may mitigate Fcγ receptor-medicated immune cell activation and lead to lower injection site reaction [18].

The efficacy of anti-TNF antibodies containing an Fc portion is reduced in patients with high titres of RF [8, 9] because the RF pentamer and anti-TNF antibody with the Fc portion form an immune complex, which may promote the clearance of the circulating anti-TNF antibody [12, 13]. Previous studies have revealed that the cut-off value for a high RF titre ranges from 166 to 204 IU/ml [10, 19]. For patients with RF titres above these values, anti-TNF inhibitors without an Fc portion, such as CZP, have demonstrated greater effectiveness than anti-TNF inhibitors with an Fc portion. The current study demonstrated that OZR exhibited effectiveness regardless of the RF titres. Our recent studies have demonstrated that patients with higher RF (>150–190 IU/ml) are at a high risk of developing difficult-to-treat RA [20, 21]. Baseline interferon α levels have been reported to correlate with RF titres in early RA and play a critical role in treatment resistance [22]. Thus, early aggressive therapy may be desirable for patients with high baseline RF levels. The current study indicated that OZR could be a good therapeutic option for these patients.

Recently, several studies have revealed that effective RA treatment decreases RF levels. For example, RF titres decreased by >40% in patients who responded well to MTX plus prednisone therapy, whereas they decreased by <10% in those who showed no response at 12 months [23]. In addition, successful drug discontinuation may be achieved when RF titres decrease after treatment [11, 24]. RF is also associated with serum IL-6 levels [25]. Consistent with this report, the present study demonstrated that RF titres, but not ACPA titres, were correlated with serum IL-6 levels at baseline. In addition, the reduction in RF titres, but not ACPA titres, from baseline to week 52 was associated with a decrease in serum IL-6 levels (Supplementary Fig. S6, available at *Rheumatology* online). These findings suggest that the decrease in serum IL-6 levels induced by OZR 30 mg may at least partially contribute to the reduction in RF titres.

Effective RA treatment also decreases ACPA titres. Misaki *et al.* showed that abatacept decreased APCA titres by ∼30% over 52 weeks in patients with a disease duration <1 year, whereas no significant change was observed in patients with a disease duration ≥1 year [26]. Atsumi *et al.* demonstrated that the combination of high-dose MTX and CZP for early RA reduced ACPA titres from an average of 176.7–128.0 U/ml after 52 weeks [27]. CZP and OZR seem to be numerically even regarding the decrease of ACPA titres, although these studies differ significantly with respect to disease duration. Further studies are warranted to elucidate the mechanisms underlying RF and ACPA titre reduction.

The NATSUZORA trial evaluated the efficacy and safety of OZR 30 mg and 80 mg for active RA without concomitant MTX [28]. The pharmacokinetics of OZR were analysed using data from the OHZORA and NATSUZORA trials [29]. The maximum plasma concentration was achieved in 6 days for both OZR 30 mg and 80 mg, and the elimination half-life was estimated to be 18 days. The receiver operating characteristic analysis yielded a cut-off trough concentration of 1 μg/ml at week 16 [29]. Our results indicated that although small differences were present in plasma concentrations depending on the RF and ACPA values, and that the mechanism underlying the lower plasma OZR concentrations in patients with high ACPA titres remains unclear, effective trough concentrations were achieved in all the groups and were maintained for up to 52 weeks (Supplementary Fig. S9, available at *Rheumatology* online).

The generation of new anti-OZR antibodies or an increased level of existing anti-OZR response was observed in 30.8% and 29.2% of the OZR 30 mg and 80 mg groups, respectively; however, the presence of the antibodies was not associated with efficacy or safety, regardless of the dose [7]. Neutralizing antibodies were confirmed in 7.0% and 5.2% of the OZR 30 mg and 80 mg groups, respectively. Neutralizing antibodies are frequently associated with secondary failure because they neutralize the effects of bDMARDs and promote their clearance [30]. However, the therapeutic response to OZR in patients with neutralizing antibodies was comparable to that in those without neutralizing antibodies in the OHZORA trial [7]. The current study demonstrated that RF and ACPA titres did not affect the production of neutralizing antibodies.

The current study has several limitations. First, this was a retrospective analysis of the OHZORA trial conducted in Japan. Further prospective studies including multiethnic populations are needed to validate these findings. Second, the OHZORA trial examined the efficacy of OZR in combination with MTX. Hence, whether OZR exhibits effectiveness regardless of RF and ACPA titres without concomitant MTX remains unclear. Additionally, the median MTX dose in this study was 10 mg/week, which is lower than the doses commonly used in other countries. Third, the LOCF method was used for missing data, including discontinuations. Various methods for data imputation exist; however, the method adopted in the OHZORA trial was used in the current study. Fourth, the underlying mechanism by which OZR treatment decreases RF and ACPA titres remains unclear. Finally, the current study included 143 patients who remained on OZR 30 mg for 52 weeks. Thus, nine patients who were initially assigned to OZR 30 mg but were reallocated to 80 mg because of the early escape criteria were excluded. However, when a total of 152 patients were analysed, including those nine, the results remained largely unchanged (Supplementary Fig. S8, available at *Rheumatology* online).

Despite these limitations, this study demonstrated that OZR, a novel NANOBODY^®^ anti-TNF inhibitor, shows effectiveness regardless of RF and ACPA titres. OZR could be an optimal drug in patients with inadequate response to MTX, particularly with high autoantibody titres.

## Supplementary Material

keaf171_Supplementary_Data

## Data Availability

The dataset used and analysed in the current study are available from the corresponding author upon reasonable request.
