# Supplementary material for: Ozoralizumab shows effectiveness regardless of baseline RF and ACPA titres in patients with RA: a post hoc analysis of the OHZORA trial
Source: Rheumatology (Oxford). 2025 Mar 26;64(7):4190–9. doi: 10.1093/rheumatology/keaf171 (PMC12212905; doi:10.1093/rheumatology/keaf171)
Supplement: keaf171_Supplementary_Data [file keaf171_supplementary_data.zip › keaf171_Supplementary_Data/rhe-24-2687-File008.docx]

**Supplementary Table S1.** **Baseline characteristics of 381 patients classified into four groups based on rheumatoid factor titres.**

| Variable | RF1 (3≤ RF <20) | RF2 (20≤ RF <49) | RF3 (49≤ RF <152) | RF4 (152≤ RF) | P Value |
| --- | --- | --- | --- | --- | --- |
| N | 92 | 97 | 95 | 97 |  |
| Age (years) | 55.00 [45.00, 62.25] | 55.00 [44.00, 61.00] | 58.00 [47.50, 67.00] | 58.00 [49.00, 64.00] | 0.033 |
| Female sex (%) | 66 (71.7) | 77 (79.4) | 75 (78.9) | 67 (69.1) | 0.252 |
| Disease duration (years) | 3.00 [1.00, 8.25] | 5.00 [1.00, 9.00] | 7.00 [3.00, 12.00] | 7.00 [3.00, 12.00] | <0.001 |
| RF-positive (%) | 21 (22.8) | 97 (100.0) | 95 (100.0) | 97 (100.0) | <0.001 |
| RF (IU/mL) | 9.00 [3.00, 15.00] | 29.00 [23.00, 37.00] | 76.00 [61.00, 106.50] | 298.00 [198.00, 485.00] | <0.001 |
| ACPA-positive (%) | 49 (53.3) | 92 (94.8) | 90 (94.7) | 96 (99.0) | <0.001 |
| ACPA (U/mL) | 5.70 [0.50, 86.55] | 138.00 [35.00, 421.00] | 134.00 [34.00, 481.00] | 241.00 [83.50, 822.00] | <0.001 |
| Seropositive (%) | 52 (56.5) | 97 (100.0) | 95 (100.0) | 97 (100.0) | <0.001 |
| RF & ACPA-positive (%) | 18 (19.6) | 92 (94.8) | 90 (94.7) | 96 (99.0) | <0.001 |
| ESR (mm/h) | 29.00 [20.00, 39.25] | 32.00 [20.00, 45.00] | 39.00 [27.00, 51.00] | 42.00 [30.00, 58.00] | <0.001 |
| hsCRP (mg/dL) | 0.70 [0.28, 1.66] | 0.74 [0.17, 2.04] | 0.74 [0.30, 1.51] | 0.72 [0.34, 1.91] | 0.711 |
| IL-6 (pg/mL) | 14.50 [5.25, 39.25] | 15.20 [3.96, 52.30] | 18.50 [6.05, 56.90] | 24.80 [7.17, 66.30] | 0.085 |
| MMP-3 (ug/L) | 133.65 [82.93, 251.25] | 139.90 [68.60, 260.60] | 137.40 [67.05, 219.40] | 152.30 [79.30, 245.30] | 0.601 |
| SJC28 | 8.00 [6.00, 11.00] | 8.00 [7.00, 11.00] | 9.00 [7.00, 11.00] | 10.00 [7.00, 15.00] | 0.01 |
| TJC28 | 8.00 [6.00, 12.25] | 9.00 [7.00, 13.00] | 9.00 [6.00, 13.00] | 12.00 [9.00, 15.00] | 0.005 |
| Pain VAS (mm) | 52.00 [23.00, 74.00] | 50.00 [24.00, 72.00] | 48.00 [34.00, 74.50] | 59.00 [31.00, 81.00] | 0.174 |
| Ph-GA (mm) | 62.00 [45.50, 77.25] | 61.00 [40.75, 72.25] | 59.00 [42.00, 75.50] | 67.00 [49.00, 78.00] | 0.183 |
| Pt-GA (mm) | 53.00 [28.00, 75.25] | 51.00 [28.25, 76.00] | 50.00 [33.50, 76.50] | 58.00 [34.00, 83.00] | 0.379 |
| DAS28-CRP | 4.92 [4.30, 5.71] | 4.95 [4.44, 5.85] | 5.10 [4.34, 5.69] | 5.40 [4.64, 6.07] | 0.025 |
| DAS28-ESR | 5.49 [4.95, 6.18] | 5.66 [4.97, 6.44] | 5.90 [5.19, 6.44] | 6.26 [5.52, 6.77] | <0.001 |
| CDAI | 30.20 [23.30, 36.50] | 28.80 [22.80, 36.85] | 29.10 [24.00, 35.15] | 36.30 [26.90, 43.70] | 0.001 |
| SDAI | 31.06 [24.13, 39.58] | 29.43 [23.84, 38.64] | 31.27 [24.49, 38.12] | 37.27 [28.17, 45.78] | 0.001 |
| HAQ-DI | 0.88 [0.38, 1.38] | 1.00 [0.38, 1.38] | 1.00 [0.50, 1.50] | 1.25 [0.75, 1.62] | 0.049 |
| MTX (mg/week) | 10.00 [8.00, 12.00] | 10.00 [8.00, 12.00] | 10.00 [8.00, 12.00] | 10.00 [8.00, 12.00] | 0.427 |
| GC use (%) | 48 (52.2) | 37 (38.1) | 38 (40.0) | 40 (41.2) | 0.207 |
| Prior biologics use (%) | 27 (29.3) | 26 (26.8) | 40 (42.1) | 39 (40.2) | 0.059 |

Results are expressed as median [interquartile range] for continuous variables or the number (%) for nominal variables. Prior biologics use includes prior use of any biologic and/or targeted synthetic disease-modifying antirheumatic drugs.

ACPA, anti-citrullinated peptide antibody; CDAI, Clinical Disease Activity Index; CRP, C-reactive protein; DAS28-CRP, disease activity score using C-reactive protein; DAS28-ESR, disease activity score using erythrocyte sedimentation rate; ESR, erythrocyte sedimentation rate; GC, glucocorticoid; HAQ-DI, Health Assessment Questionnaire Disability Index; hsCRP, high-sensitivity C-reactive protein; IL-6, interleukin-6; MMP-3, matrix metalloproteinase-3; MTX, methotrexate; Ph-GA, physicians’ global assessment; Pt-GA, patients’ global assessment; RF, rheumatoid factor; SDAI, Simplified Disease Activity Index; SJC28, swollen joint count of 28 joints; TJC28, tender joint count of 28 joints; VAS, visual analogue scale.

**Supplementary Table S2. Baseline characteristics of 381 patients classified into four groups based on anti-citrullinated peptide antibody titres.**

| Variable | ACPA1  (0.5≤ ACPA<25.9) | ACPA2  (25.9 ≤ ACPA<103) | ACPA3  (103≤ ACPA<426) | ACPA4  (426≤ ACPA) | P Value |
| --- | --- | --- | --- | --- | --- |
| N | 95 | 94 | 96 | 96 |  |
| Age (years) | 55.00 [44.00, 64.00] | 55.00 [48.00, 61.75] | 55.00 [46.00, 61.25] | 59.00 [51.75, 66.00] | 0.045 |
| Female sex (%) | 67 (70.5) | 81 (86.2) | 71 (74.0) | 66 (68.8) | 0.026 |
| Disease duration (years) | 5.00 [1.00, 9.00] | 7.00 [2.00, 11.00] | 5.00 [3.00, 11.00] | 6.00 [1.00, 11.25] | 0.285 |
| RF-positive (%) | 47 (49.5) | 84 (89.4) | 86 (89.6) | 93 (96.9) | <0.001 |
| RF (IU/mL) | 15.00 [3.00, 37.50] | 61.00 [22.25, 149.25] | 61.00 [25.00, 178.75] | 71.50 [35.50, 281.75] | <0.001 |
| ACPA-positive (%) | 41 (43.2) | 94 (100.0) | 96 (100.0) | 96 (100.0) | <0.001 |
| ACPA (U/mL) | 1.90 [0.50, 10.85] | 51.95 [35.00, 69.58] | 193.00 [140.50, 323.25] | 911.00 [570.25, 1200.00] | <0.001 |
| Seropositive (%) | 55 (57.9) | 94 (100.0) | 96 (100.0) | 96 (100.0) | <0.001 |
| RF & ACPA-positive (%) | 33 (34.7) | 84 (89.4) | 86 (89.6) | 93 (96.9) | <0.001 |
| ESR (mm/h) | 32.00 [21.50, 38.50] | 35.50 [26.00, 50.00] | 37.00 [23.75, 50.25] | 40.50 [26.75, 53.00] | 0.009 |
| hsCRP (mg/dL) | 0.60 [0.18, 1.33] | 0.86 [0.40, 1.83] | 0.62 [0.20, 1.74] | 0.81 [0.29, 2.31] | 0.074 |
| IL-6 (pg/mL) | 13.00 [4.17, 44.20] | 16.80 [6.68, 42.17] | 17.20 [6.09, 67.42] | 25.95 [7.25, 66.23] | 0.112 |
| MMP-3 (ug/L) | 126.60 [57.35, 228.75] | 145.45 [81.52, 225.73] | 146.95 [80.95, 237.62] | 151.75 [81.58, 247.07] | 0.368 |
| SJC28 | 8.00 [7.00, 13.00] | 8.00 [7.00, 12.00] | 9.00 [7.00, 11.00] | 9.00 [7.00, 11.00] | 0.945 |
| TJC28 | 9.00 [6.00, 14.00] | 9.50 [7.00, 13.00] | 10.00 [7.00, 14.00] | 10.00 [7.00, 15.00] | 0.533 |
| Pain VAS (mm) | 42.00 [22.50, 71.00] | 46.00 [25.25, 74.00] | 52.00 [30.75, 78.25] | 61.00 [37.00, 77.25] | 0.019 |
| Ph-GA (mm) | 63.50 [44.75, 77.25] | 59.50 [42.00, 71.75] | 61.00 [43.75, 76.25] | 65.00 [48.25, 76.00] | 0.697 |
| Pt-GA (mm) | 48.50 [25.25, 75.00] | 48.50 [29.00, 69.75] | 58.00 [30.75, 79.25] | 60.00 [42.75, 80.00] | 0.045 |
| DAS28-CRP | 5.00 [4.18, 5.73] | 5.10 [4.44, 5.68] | 4.99 [4.37, 5.75] | 5.25 [4.68, 6.10] | 0.053 |
| DAS28-ESR | 5.59 [4.92, 6.40] | 5.68 [5.21, 6.46] | 5.72 [5.15, 6.51] | 6.06 [5.35, 6.77] | 0.043 |
| CDAI | 31.90 [22.60, 41.20] | 28.70 [23.20, 37.40] | 31.00 [24.40, 37.25] | 32.80 [24.50, 38.65] | 0.373 |
| SDAI | 32.64 [23.94, 41.75] | 29.93 [24.31, 38.25] | 32.68 [25.04, 38.61] | 34.90 [26.07, 42.87] | 0.163 |
| HAQ-DI | 0.88 [0.38, 1.38] | 1.12 [0.53, 1.38] | 0.88 [0.50, 1.50] | 1.13 [0.62, 1.50] | 0.145 |
| MTX (mg/week) | 10.00 [8.00, 12.00] | 10.00 [8.00, 12.00] | 10.00 [8.00, 12.00] | 10.00 [8.00, 12.00] | 0.721 |
| GC use (%) | 37 (38.9) | 42 (44.7) | 43 (44.8) | 41 (42.7) | 0.833 |
| Prior biologics use (%) | 34 (35.8) | 37 (39.4) | 31 (32.3) | 30 (31.2) | 0.636 |

Results are expressed as median [interquartile range] for continuous variables or the number (%) for nominal variables. Prior biologics use includes prior use of any biologic and/or targeted synthetic disease-modifying antirheumatic drugs.

ACPA, anti-citrullinated peptide antibody; CDAI, Clinical Disease Activity Index; CRP, C-reactive protein; DAS28-CRP, disease activity score using C-reactive protein; DAS28-ESR, disease activity score using erythrocyte sedimentation rate; ESR, erythrocyte sedimentation rate; GC, glucocorticoid; HAQ-DI, Health Assessment Questionnaire Disability Index; hsCRP, high-sensitivity C-reactive protein; IL-6, interleukin-6; MMP-3, matrix metalloproteinase-3; MTX, methotrexate; Ph-GA, physicians’ global assessment; Pt-GA, patients’ global assessment; RF, rheumatoid factor; SDAI, Simplified Disease Activity Index; SJC28, swollen joint count of 28 joints; TJC28, tender joint count of 28 joints; VAS, visual analogue scale.

**Supplementary Table S3. Multivariable logistic regression analysis for DAS28-CRP remission.**

|  | Model 1 | | | | Model 2 | | | | |
| --- | --- | --- | --- | --- | --- | --- | --- | --- | --- |
|  | Odds ratio | 95%CI  lower | 95%CI  upper | *P* value |  | Odds ratio | 95%CI  lower | 95%CI  upper | *P* value |
| RF titre group | 0.975 | 0.691 | 1.38 | 0.885 | RF titre reduction | 1.00 | 0.998 | 1.00 | 0.644 |
| ACPA titre group | 0.774 | 0.548 | 1.09 | 0.147 | ACPA titre reduction | 1.00 | 0.999 | 1.00 | 0.131 |
| MTX dose | 1.02 | 0.907 | 1.15 | 0.722 | MTX dose | 1.02 | 0.908 | 1.16 | 0.698 |
| Prior biologics use | 0.982 | 0.468 | 2.06 | 0.961 | Prior biologics use | 0.975 | 0.474 | 2.01 | 0.945 |

ACPA, anti-citrullinated peptide antibody; CI, confidence interval: DAS28-CRP, disease activity score using C-reactive protein; MTX, methotrexate; RF, rheumatoid factor.
