# Supplementary material for: Ozoralizumab shows effectiveness regardless of baseline RF and ACPA titres in patients with RA: a post hoc analysis of the OHZORA trial
Source: Rheumatology (Oxford). 2025 Mar 26;64(7):4190–9. doi: 10.1093/rheumatology/keaf171 (PMC12212905; doi:10.1093/rheumatology/keaf171)
Supplement: keaf171_Supplementary_Data [file keaf171_supplementary_data.zip › keaf171_Supplementary_Data/rhe-24-2687-File009.docx]

**Supplementary Figure S1.**


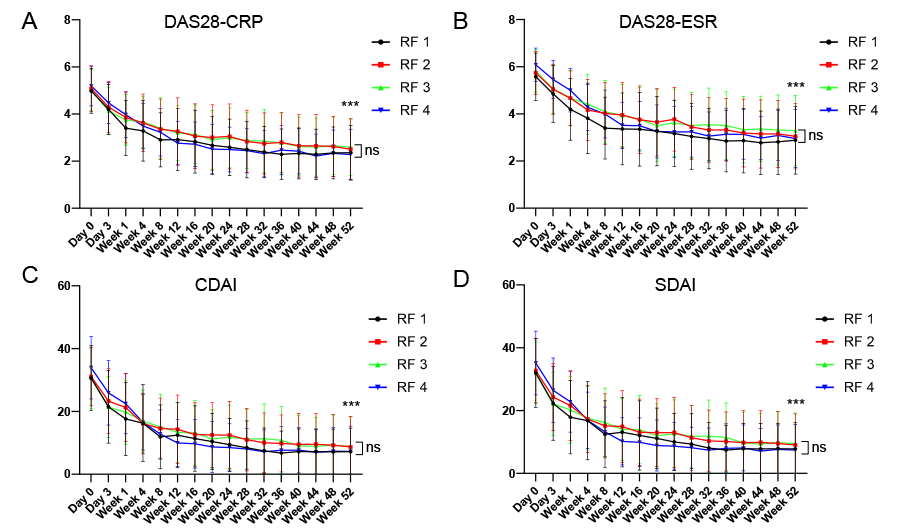


**Ozoralizumab (OZR) 80 mg effectively controlled disease activities over 52 weeks irrespective of baseline RF titres.**

A total of 154 patients who received OZR 80 mg over 52 weeks were classified into four groups based on the baseline RF titre quartiles (RF1: RF 3–20 IU/mL, RF2: 20–49 IU/mL, RF3: 49–153 IU/mL, RF4: 153–2029 IU/mL), and changes in (A) DAS28-CRP, (B) DAS28-ESR, (C) CDAI, and (D) SDAI were shown. Data are shown as mean±SD. The last observation carried forward method was used.

CDAI, Clinical Disease Activity Index; DAS28-CRP, disease activity score using C-reactive protein; DAS28-ESR, disease activity score using erythrocyte sedimentation rate; ns, not significant; RF, rheumatoid factor; SDAI, Simplified Disease Activity Index. *** *P*<0.001

**Supplementary Figure S2.**


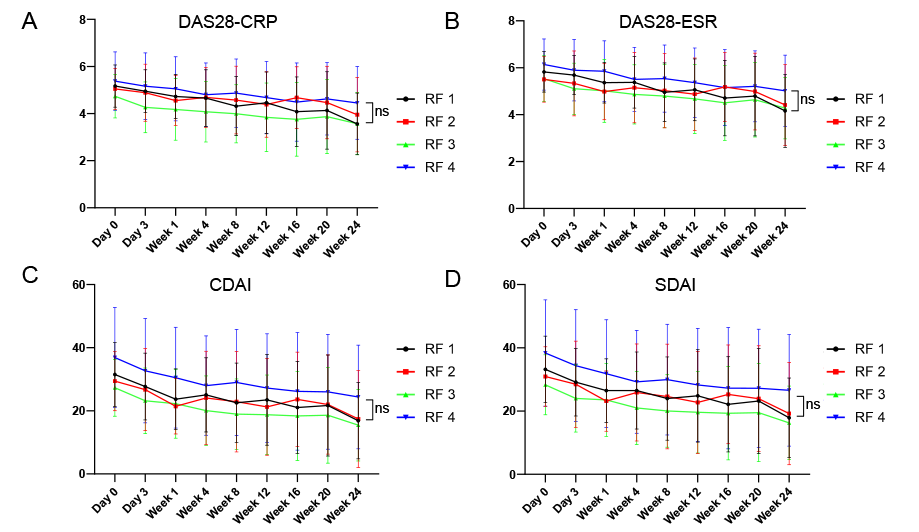


**Placebo slowly decreased disease activities over 24 weeks.**

A total of 75 patients who received placebo over 24 weeks were classified into four groups based on the baseline RF titre quartiles (RF1: RF 3–20 IU/mL, RF2: 20–49 IU/mL, RF3: 49–153 IU/mL, RF4: 153–2029 IU/mL), and changes in (A) DAS28-CRP, (B) DAS28-ESR, (C) CDAI, and (D) SDAI were shown. Data are shown as mean±SD. The last observation carried forward method was used.

CDAI, Clinical Disease Activity Index; DAS28-CRP, disease activity score using C-reactive protein; DAS28-ESR, disease activity score using erythrocyte sedimentation rate; ns, not significant; RF, rheumatoid factor; SDAI, Simplified Disease Activity Index.

**Supplementary Figure S3.**

**
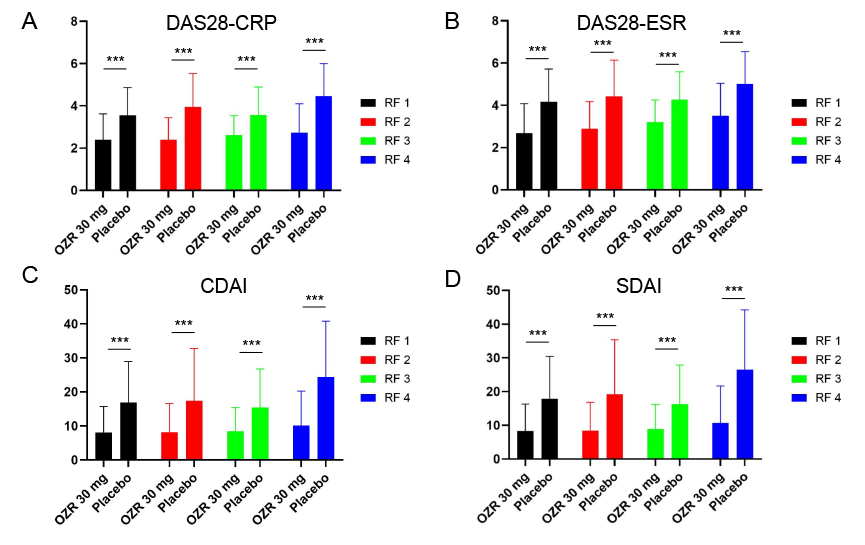
**

**A comparison of disease activity at week 24 between ozoralizumab (OZR) 30 mg and placebo in each rheumatoid factor (RF) titre group.**

Disease activity at week 24 was compared between 143 patients who received OZR 30mg and 75 patients who received placebo in each RF titre group (RF1: RF 3–20 IU/mL, RF2: 20–49 IU/mL, RF3: 49–153 IU/mL, RF4: 153–2029 IU/mL). (A) DAS28-CRP, (B) DAS28-ESR, (C) CDAI, and (D) SDAI were shown. CDAI, Clinical Disease Activity Index; DAS28-CRP, disease activity score using C-reactive protein; DAS28-ESR, disease activity score using erythrocyte sedimentation rate; SDAI, Simplified Disease Activity Index. *** *P*<0.001

**Supplementary Figure S4.**


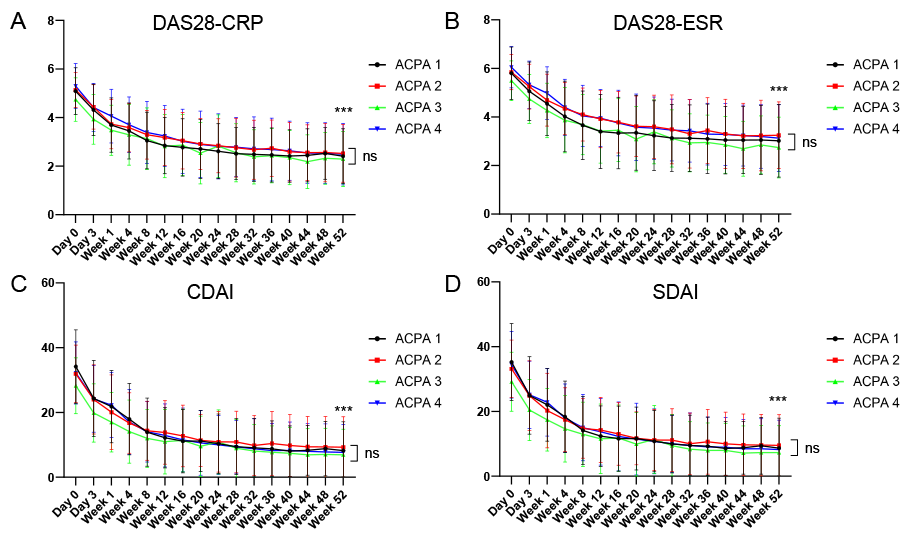


**Ozoralizumab (OZR) 80 mg effectively controlled disease activities over 52 weeks irrespective of baseline anti-citrullinated peptide antibody (ACPA) titres.**

A total of 154 patients who received OZR 80 mg over 52 weeks were classified into four groups based on the baseline ACPA titre quartiles (ACPA1: 0.5–25.9 U/mL, ACPA2: 25.9–103 U/mL, ACPA3: 103–426 U/mL, ACPA4: 426–1200 U/mL), and changes in (A) DAS28-CRP, (B) DAS28-ESR, (C) CDAI, and (D) SDAI were shown. Data are shown as mean±SD. The last observation carried forward method was used.

CDAI, Clinical Disease Activity Index; DAS28-CRP, disease activity score using C-reactive protein; DAS28-ESR, disease activity score using erythrocyte sedimentation rate; ns, not significant; SDAI, Simplified Disease Activity Index. *** *P*<0.001

**Supplementary Figure S5.**


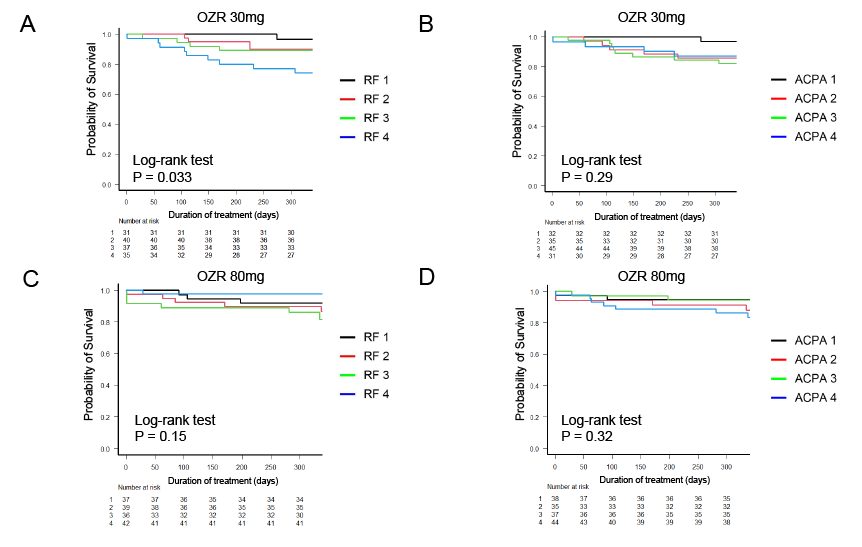


**Drug retention rates of ozoralizumab (OZR).**

The drug retention rates of OZR 30mg were examined based on the baseline (A) rheumatoid factor (RF) and (B) anti-citrullinated peptide antibody (ACPA) titres. The drug retention rates of OZR 80 mg were also examined based on the baseline (C) RF and (D) ACPA titres. The baseline RF titre quartiles are as follows; RF1: RF 3–20 IU/mL, RF2: 20–49 IU/mL, RF3: 49–153 IU/mL, RF4: 153–2029 IU/mL. The baseline ACPA titre quartiles are as follows; ACPA1: 0.5–25.9 U/mL, ACPA2: 25.9–103 U/mL, ACPA3: 103–426 U/mL, ACPA4: 426–1200 U/mL.

**Supplementary Figure S6.**


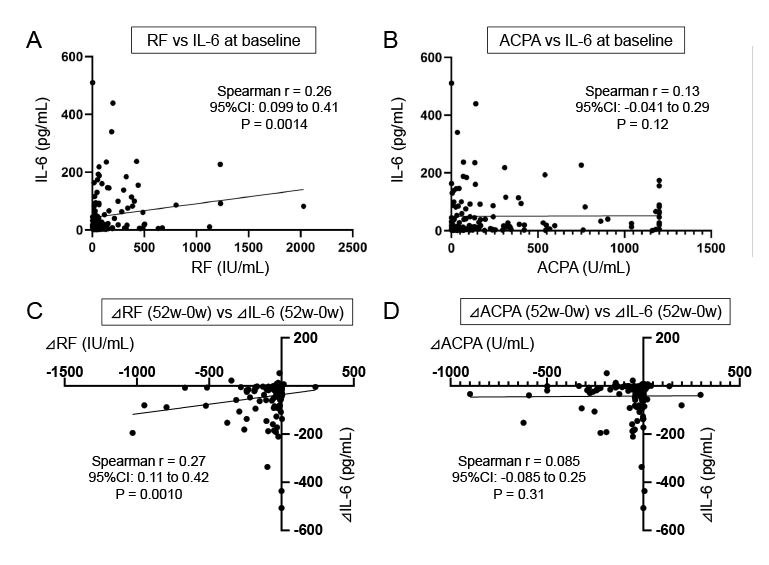


**Correlations between rheumatoid factor (RF) and anti-citrullinated peptide antibody (ACPA) titres and serum interleukin (IL)-6 levels.**

Correlations between RF and ACPA titres and serum IL-6 levels were examined in 143 patients who received ozoralizumab 30 mg over 52 weeks. (A) Correlation between RF titres and IL-6 levels at baseline, (B) correlation between ACPA titres and IL-6 levels at baseline, (C) correlation between the reduction in RF titres and IL-6 levels from baseline to week 52, and (D) correlation between the reduction in ACPA titres and IL-6 levels from baseline to week 52. Correlation was analysed using Spearman’s correlation analysis. CI, confidence interval.

**Supplementary Figure S7.**


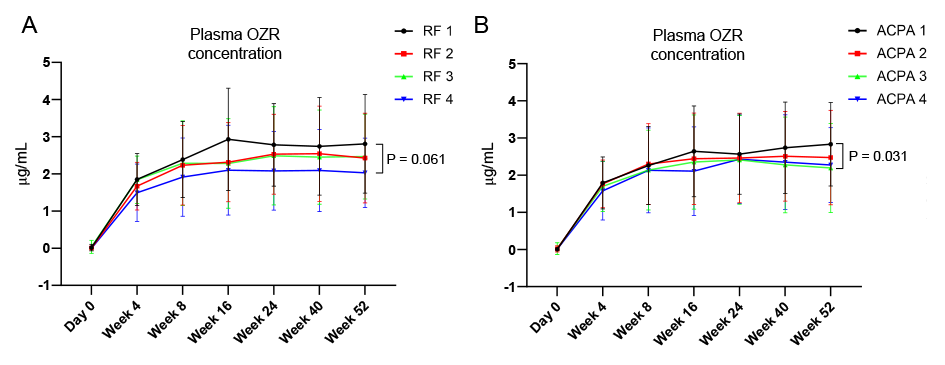


**Time course of plasma ozoralizumab (OZR) concentrations.**

A total of 143 patients who received OZR 30 mg over 52 weeks were classified into four groups based on (A) the baseline rheumatoid factor (RF) titre quartiles (RF1: RF 3–20 IU/mL, RF2: 20–49 IU/mL, RF3: 49–153 IU/mL, RF4: 153–2029 IU/mL) or (B) the baseline anti-citrullinated peptide antibody (ACPA) titre quartiles (ACPA1: 0.5–25.9 U/mL, ACPA2: 25.9–103 U/mL, ACPA3: 103–426 U/mL, ACPA4: 426–1200 U/mL). Time course of plasma OZR concentrations in each (A) RF group and (B) ACPA group were shown. Data are shown as mean±SD.

**Supplementary Figure S8.**


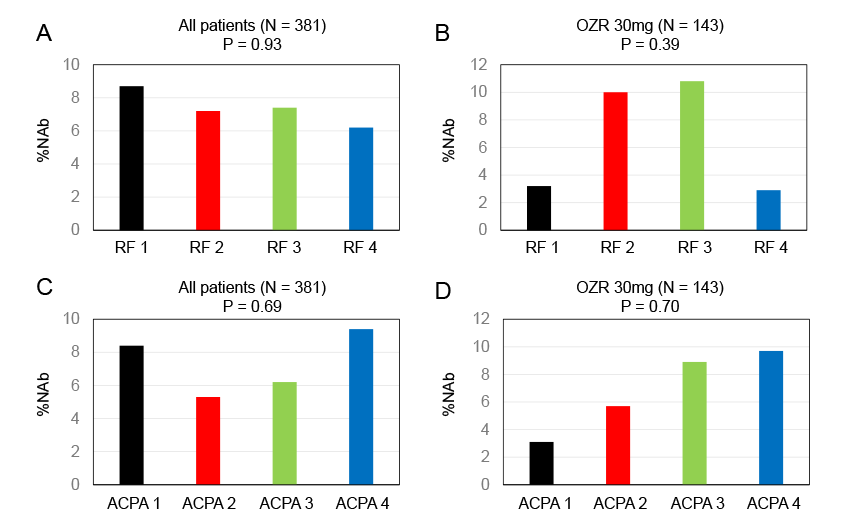


**Plasma ozoralizumab (OZR)-neutralizing antibodies.**

Neutralizing antibodies against OZR were examined based on the baseline rheumatoid factor (RF) and anti-citrullinated peptide antibody (ACPA) titres.

The prevalence of OZR- neutralizing antibodies in (A) all patients sorted by RF titres, (B) 143 patients treated with OZR 30 mg sorted by RF titres, (C) all patients sorted by ACPA titres, and (D) 143 patients treated with OZR 30 mg sorted by ACPA titres. The RF titre quartiles are as follows; RF1: RF 3–20 IU/mL, RF2: 20–49 IU/mL, RF3: 49–153 IU/mL, RF4: 153–2029 IU/mL. The ACPA titre quartiles are as follows; ACPA1: 0.5–25.9 U/mL, ACPA2: 25.9–103 U/mL, ACPA3: 103–426 U/mL, ACPA4: 426–1200 U/mL.

**Supplementary Figure S9.**


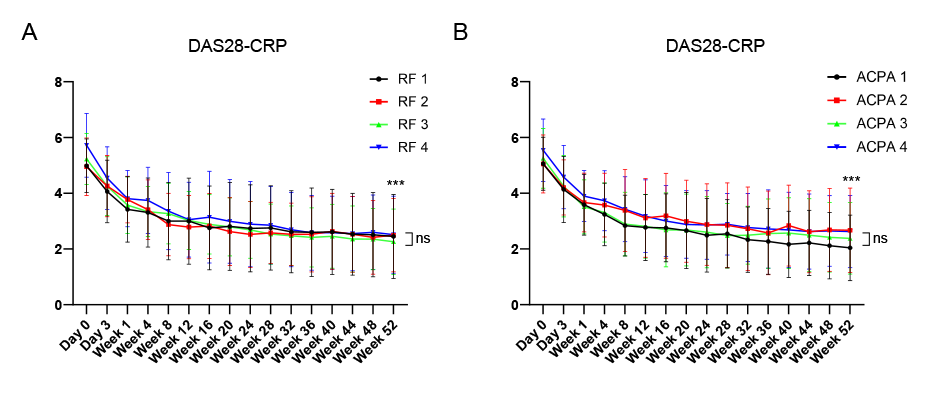


**Ozoralizumab (OZR) 30 mg effectively controlled disease activities irrespective of baseline RF and ACPA titres, even when 9 patients who met the early escape criteria were included.**

A total of 152 patients who were initially allocated to OZR 30 mg were classified into four groups based on (A) the baseline RF titre quartiles (RF1; n = 35, RF2; n = 42, RF3; n = 38, RF4; n = 37) or (B) the ACPA titre quartiles (ACPA1; n = 34, ACPA2; n = 40, ACPA3; n = 46, ACPA4; n = 32), and changes in DAS28-CRP were shown. In the nine patients who met the early escape criteria, DAS28-CRP at 24 weeks and beyond was substituted for those at 20 weeks before OZR 80 mg was initiated. Data are shown as mean±SD. *** *P*<0.001. The baseline RF titre quartiles are as follows; RF1: RF 3–20 IU/mL, RF2: 20–49 IU/mL, RF3: 49–153 IU/mL, RF4: 153–2029 IU/mL. The baseline ACPA titre quartiles are as follows; ACPA1: 0.5–25.9 U/mL, ACPA2: 25.9–103 U/mL, ACPA3: 103–426 U/mL, ACPA4: 426–1200 U/mL. The last observation carried forward method was used.

DAS28-CRP, disease activity score using C-reactive protein; ns, not significant; RF, rheumatoid factor.
